# A dual-gene-deleted ASFV Lv17/WB/Rie1-ΔCD candidate administered orally to wild boar confers DIVA-compatible protection against virulent challenge

**DOI:** 10.1080/01652176.2026.2649573

**Published:** 2026-03-26

**Authors:** Jose A. Barasona, Aleksandra Kosowska, Gabriela González-García, Marta Díaz-Frutos, Giulia Franzoni, Paola Nicolussi, Nestor Porras, Mónica Sánchez-Segovia, Daniel De Antonio-Gómez, Paloma Rueda, Sandra Barroso-Arévalo

**Affiliations:** a Animal Health Department, Faculty of Veterinary Medicine, Complutense University of Madrid, Madrid, Spain; b VISAVET Health Surveillance Center, Complutense University of Madrid, Madrid, Spain; c Gold Standard Diagnostics Madrid SA (GSD Madrid), Madrid, Spain; d Istituto Zooprofilattico Sperimentale della Sardegna, Sassari, Italy; e Department of Animal Medicine and Surgery, Faculty of Veterinary Medicine, Complutense University of Madrid, Madrid, Spain

**Keywords:** African swine fever virus, oral vaccination, live attenuated vaccine, wild boar, DIVA properties

## Abstract

African swine fever (ASF) continues to expand worldwide. Recent detection in wild boar in Spain highlights the urgent need for effective control tools, with oral vaccination as a key priority. Following previous evaluation of the attenuated Lv17/WB/Rie1 strain, we assess an improved derivative, Lv17/WB/Rie1-ΔCD, lacking EP402R (CD2v) and EP153R, replaced by GFP to abrogate haemadsorption and enable Differentiating-Infected-from-Vaccinated-Animals (DIVA) diagnostics. Vaccinated animals received either a single high dose (10^4^ TCID₅₀) or a prime and re-exposure regimen (10^2^ TCID₅₀ plus a 10^4^ TCID₅₀). Animals were challenged intramuscularly with the virulent Armenia07 genotype II strain. The ΔCD vaccine was well tolerated, inducing only transient low-grade fever. Prime–re-exposure vaccination induced earlier seroconversion (mean 12 ± 4 dpv) and sterilizing immunity in 5/6 animals in the high dose group. Overall protection reached 90%, while all unvaccinated controls died within 7 days. Quantitative PCR revealed >10³-fold reductions in viral genome copies in blood and tissues versus controls. DIVA ELISA reliably distinguished vaccine-induced antibodies from infection-derived responses. These findings identify Lv17/WB/Rie1-ΔCD as a safer oral ASFV vaccine candidate, addressing concerns raised with the parental Lv17/WB/Rie1 by increasing attenuation and supporting multi-gene deletion strategies. Further studies on safety, transmission, genetic stability, and environmental behaviour are required before large-scale field trials.

## Introduction

African swine fever (ASF) is a highly contagious and deadly disease that affects pigs and wild boars, with the latter playing a key role in its transmission (Podgórski and Śmietanka 2018). ASF remains a persistent challenge in many affected countries, threatening global food security and biodiversity, particularly as pork accounts for more than 35% of global meat consumption. Wild boar (*Sus scrofa ferus*) populations, especially in the EU, are central to virus spread, disrupting the ecosystem balance and complicating control efforts. The disease is caused by the African swine fever virus (*Asfivirus haemorragiae;* ASFV), the sole member of the *Asfarviridae* family belonging to the *Asfavirus* genus. ASFV is capable of causing severe haemorrhagic disease in pigs of all ages, with lethality rates of up to 100% (Arias and Sánchez-Vizcaíno 2012; Alonso et al. 2018). The clinical signs of the disease vary depending on the strain of the virus and the susceptibility of the host. In acute cases, the onset of clinical signs can be sudden, with high fever, loss of appetite and death within a few days (Sánchez-Vizcaíno et al. 2015). In sub-acute and chronic cases, the clinical signs can include fever, anaemia, weight loss, skin discoloration, and internal haemorrhages (Arias et al. 2002). The virus can be transmitted through direct contact, contaminated feed and water, and wild boar populations that act as reservoirs (Guinat et al. 2016).

For all these reasons, the disease causes significant economic losses in the pork industry and poses a threat to food security. ASF has expanded globally over the last decade, becoming a major transboundary animal disease (TAD) with devastating effects in endemic regions. Since its introduction in Georgia in 2007, ASFV has spread across Europe, Asia, and Africa, and in 2021, it was detected in the Dominican Republic and Haiti, marking its re-entry into the Americas after decades of absence (FAO 2025; Schambow et al. 2025). By late 2024, it was confirmed for the first time in Greece and Sweden (EFSA 2024). Most recently, in November 2025, ASF was officially reported in wild boars in a region of Catalonia in northeastern Spain, highlighting the continued epidemiological risk and the need for reinforced surveillance and control strategies. In addition, ASFV can survive for long periods in the environment, and it can be found in pork products, animal by-products, soil, and water (Olesen et al. 2020). This makes it difficult to control the spread of the disease, especially in wild boar populations, where it can persist and disseminate through the movement of the animals. The virus is also able to cross international borders through the illegal trade of pork products and the movement of infected wild boars. Although some countries have successfully eradicated ASF through widespread culling and improved biosecurity measures (Danzetta et al. 2020), these methods alone have proven insufficient for long-term control. Vaccination is now increasingly recognized as an efficient tool for reducing the susceptible population, limiting virus transmission, and minimizing the need for large-scale culling (Gortazar et al. 2015). This approach stabilizes pork supply chains and fosters support from stakeholders such as hunters and citizen scientists engaged in passive surveillance.

The control of ASF has been hindered by a lack of effective vaccines and inherent difficulties in managing the disease in complex environments. Current control measures, such as culling and quarantine, have been implemented in domestic pigs but have not been successful in controlling the disease in wild boars. Wild boars have been identified as major drivers of ASFV persistence in both endemic and epidemic regions, acting as long-term reservoirs for the virus and complicating eradication efforts (Pepin et al. 2020). The development of an ASF vaccine remains highly challenging due to the complexity of ASFV, which encodes over 160 polypeptides, many specialized in evading immune responses (Dixon et al. 2004). The virus's genetic diversity and its ability to infect monocytes and macrophages, while employing poorly understood immune evasion mechanisms, have further complicated control efforts. Evidence nonetheless suggests that both cellular and serological immunity play a role in protection against ASFV (Takamatsu et al. 2013; Schäfer et al. 2022).

Several studies have been conducted to develop a vaccine against ASF, with different approaches and types of vaccines used. One approach that has been studied is the use of inactivated virus vaccines (Cadenas-Fernández et al. 2021). Inactivated ASFV preparations have repeatedly failed to provide solid protection despite inducing antibody responses (Bosch-Camós et al. 2020; Vu and McVey 2024). Additionally, it can be difficult to inactivate the virus without altering its antigenicity, which can affect the effectiveness of the vaccine. Another approach is the use of virus-vectored vaccines, which are made by expressing the ASFV antigen in a different virus, such as a poxvirus, that can infect the animal and induce an immune response (Urbano and Ferreira 2022). These vaccines have not proven to be effective enough as vaccine prototypes. Recent advances have focused on modified live vaccines (MLVs) through the deletion of virulence-associated genes, with some candidates showing potential in providing complete protection against homologous lethal field strains (Zhang et al. 2023; Urbano and Ferreira 2022; Vu and McVey 2024). The introduction of the first commercial ASF vaccines in Vietnam, ASFV-G-ΔI177L and ASFV-G-ΔMGF, has raised hopes for a globally licensed product (Tran et al. 2022; Borca et al. 2020a). However, the safety profile of these candidates remains a subject of intense evaluation. For instance, cell-culture adapted variants of ASFV-G-ΔI177L have shown promising characteristics in experimental settings; nevertheless, significant safety concerns persist regarding the stability of MLVs, including reports of reversion to virulence of the ASFV-G-ΔI177L strain in pregnant sows (van den Born et al. 2025). Recent evidence from Vietnam has described the detection of an ASFV vaccine-like variant with multiple gene deletions in the field (Nguyen et al. 2025). Notably, the authors refer to a virus genetically resembling ASFV-G-ΔMGF vaccine-like variants rather than the direct administration of a commercial vaccine under controlled conditions. This finding underscores the importance of maintaining robust biosecurity and molecular surveillance when evaluating and deploying live attenuated ASFV vaccine candidates.

Developing deletion mutants based on naturally attenuated virus isolates offers a promising strategy to mitigate reversion risk and enhance vaccine safety for global ASF control. However, challenges remain, particularly in wildlife vaccination, where inconsistent uptake among free-ranging wild boars complicates predictions of their effectiveness (Cadenas-Fernández et al. 2024). Additionally, implementing such programs requires significant investment, infrastructure, and monitoring to ensure coverage and track disease progression, adding complexity and cost to ASF control efforts.

Following this latter concept, the Lv17/WB/Rie1 strain is an attenuated strain of ASF virus that has been previously evaluated as a vaccine candidate in both domestic pigs and wild boar (Gallardo et al. 2019; Barasona et al. 2019, 2021). Identified in Latvia in 2017, this naturally attenuated ASFV genotype II isolate achieved 92% protection in wild boar through oral immunization (Barasona et al. 2019; Cadenas-Fernández et al. 2024). Despite its efficacy, it exhibited safety limitations in domestic pigs, causing peaks of fever, transient lethargy, joint swelling, and occasional chronic ASF (Gallardo et al. 2019). These studies have also shown promising results in terms of inducing a strong protection against challenge with the virulent virus Armenia07 (Arm07) (Supplementary Table 1).

In addition, differentiating infected from vaccinated animals (DIVA) properties are essential for the widespread use of live attenuated vaccines, as they allow differentiation between vaccinated and infected animals. Therefore, further improvements in this attenuated strain are required to increase its safety profile and develop a DIVA test for its diagnosis. To address these issues, the Lv17/WB/Rie1-ΔCD vaccine prototype was developed, incorporating deletions of *EP153R* and *EP402R (CD2v)* genes to eliminate haemadsorption (HAD) and enable DIVA capability, substituting both genes encoding these two proteins with a reporter gene -green fluorescent protein- GFP (Patent Nº PCT/EP2023/082521) (Gonzalez-García et al. 2025). The vaccine achieved 100% seroconversion and full protection against virulent ASFV genotype II isolates in domestic pigs through intramuscular administration (Gallardo et al. 2024).

The objective of this study is to evaluate the protective efficacy of the novel deletion mutant vaccine candidate Lv17/WB/Rie1-ΔCD in wild boars via oral administration. Specifically, we aim to compare two different oral doses of the vaccine to determine their relative performance in terms of safety, immunogenicity, and protection against virulent ASFV challenge. By understanding its efficacy, safety profile, and DIVA capabilities, we hope to contribute significantly to ongoing efforts to curb the spread and impact of this devastating disease.

## Materials and methods

### Animals

Eighteen 3-month-old female wild boar piglets weighing 10–15 kg were obtained from a commercial wild boar farm in Seville, Spain. The experiment was carried out in biosafety level 3 (BSL-3) facilities at the VISAVET Health Surveillance Centre at the University Complutense of Madrid. Upon arrival, all the animals were ear-tagged for individual identification; they were first acclimated for 2 weeks before the study began. The animals had *ad libitum* access to food and water during the experimental period.

Animal care and procedures were performed in accordance with the guidelines of good experimental practices, following European, national, and regional regulations and under the supervision and approval of the Ethics Committee of Comunidad de Madrid (reference PROEX 113.3/21). The protocol included a detailed description of efforts to prevent and avoid the animals' unnecessary suffering, including humane endpoints and guidelines regarding euthanasia, following EC Directive 2010/63/UE. All procedures were designed and performed by specially trained personnel and veterinarians (animal experimentation categories B, C, and D) in accordance with Directive 2003/65/EC and Spanish regulations (RD 53/2013). Guidelines for the ARRIVE 2.0 for the care and use of laboratory animals were also followed. Animals were not sedated for routine sampling. Wild boars were manually restrained by trained personnel and specialized veterinarians using standard physical restraint techniques appropriate for the species (brief confinement and secure hold), allowing blood and swab collection to be performed rapidly and safely. Sedation was intentionally avoided to prevent potential interference with clinical observations and immune/physiological parameters.

### Experimental design

**Figure 1.**
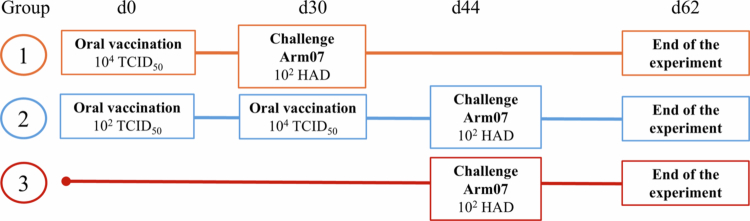
Experimental design and immunization schedule.

The study was carried out with wild boars randomly divided into two groups of six vaccinated animals each and one control group and allocated to three different pens. The three groups were housed separately in isolated containment boxes. The first group (high-dose–single administration; Group 1) received a single oral dose of 1 mL at 10⁴ TCID₅₀/mL. The second group (low-dose–high-dose re-exposure administration; Group 2) was orally vaccinated with an initial 1 mL dose at 10² TCID₅₀/mL, followed by a second-oral administration dose of 10⁴ TCID₅₀/mL (Figure 1). Both groups received the deletion mutant Lv17/WB/Rie1-ΔCD isolate. The vaccine was administered orally by depositing the suspension directly into the oral cavity using a syringe while the animals were manually restrained by experienced veterinarians. To approximate field conditions during bait consumption, mild mechanical stimulation of the oral mucosa was performed to mimic the minor abrasions that may occur during chewing, thereby facilitating mucosal exposure to the vaccine suspension.

The animals from the low-dose–high-dose re-exposure group were revaccinated with 10⁴ TCID₅₀/mL of the same isolate 30 days after the first vaccination (dpv). All groups were intramuscularly challenged with the highly virulent Arm07 virus isolate (10² HAD₅₀). The control group remained untreated during the vaccination period and was infected with the challenge isolate at day 44, together with the vaccinated Group 2, to confirm the virulence of the isolate (Figure 1).

### ASFV isolates

The dual-gene deletion (*EP402R/CD2v* and *EP153R*) used in this study corresponds to the construct previously described by Gallardo et al. (2024). Briefly, the deletion mutant was generated by CRISPR–Cas9-mediated homologous recombination, replacing both open reading frames with an enhanced green fluorescent protein (eGFP) cassette to ablate haemadsorption and provide a DIVA marker. The complete genome sequence of this construct is deposited in GenBank under accession number PQ284530 (Patent PCT/EP2023/082521). Correct deletion was confirmed by junction PCR and Sanger sequencing; the absence of a parental backbone was verified by fail-to-amplify assays targeting EP402R and EP153R (Petrini et al. 2023).

The highly virulent haemadsorbing genotype II ASFV Arm07 isolate was used as the challenge virus. This isolate shares >99.9% identity with the reference Georgia 2007/1 strain (GenBank accession number FR682468.2). Full genome characterization of the Arm07 stock used in this study has been previously described by Pérez-Núñez et al. (2020) under accession number LR812933.1. Viral stocks for inoculation were grown *in vitro* on sub-confluent monolayers of 2-day-old monocyte/macrophage cultures, and viral titres were determined as the amount of virus causing haemadsorption in 50% of inoculated cultures (HAD₅₀/mL) (WOAH Manual).

All animals vaccinated with the deletion mutant Lv17/WB/Rie1-ΔCD isolate, as well as control animals, were challenged with 10² HAD₅₀ of the ASFV Arm07 isolate administered by intramuscular inoculation at 30 dpv (high-dose and control groups) and at 44 dpv (low-dose–high-dose re-exposure group).

### Laboratory procedure

During the study period, EDTA-blood and coagulated blood for the preparation of serum were collected from each animal twice a week. Viral DNA was extracted from 200 µL of each sample using the High Pure Template Preparation Mix Kit (Roche Diagnostics GmbH, Mannheim, Germany) according to the manufacturer's instructions. The detection of ASFV DNA was performed using the Universal Probe Library (UPL) real-time PCR (qPCR) recommended by the World Organisation for Animal Health (WOAH) and previously described by Fernández-Pinero et al. (2013). Positive qPCR results were determined by identifying the threshold cycle value (Cq) at which reporter dye emission appeared above the background within 40 cycles. Positive and negative controls were used for both DNA extraction and qPCR.

Serum samples were tested for the presence of ASFV-specific antibodies using a commercial ELISA based on the detection of the VP72 ASFV antigen (INgezim® PPA Compac, ref. R.*11.PP*A.K3, Gold Standard Diagnostics, Madrid, Spain) according to the manufacturer's instructions (sensitivity: 99%, specificity: 100%; as compared to the WOAH Reference Indirect ELISA). In addition, the experimental INgezim® ASFV DIVA pEP153R ELISA was applied to differentiate antibodies induced by infection with EP153R-competent field viruses from those generated after vaccination with the EP153R-deleted Lv17/WB/Rie1-ΔCD strain. This strategy, based on the combined use of three ELISAs (INgezim® PPA Compac, pEP153R ELISA, and eGFP ELISA), was performed as previously described by González-García et al. (2025).

### Biochemical analyses

A total of 11 parameters were monitored over time during *in vivo* experimentation for comprehensive evaluation of the impact of immunization on animal health status, using previously published methods (Franzoni et al. 2026). In detail, sera were heat treated for 30 min at 56 °C, then an automated spectrophotometer (EXL 200, Siemens, Monaco, Germany) was subsequently used to quantify the levels of albumin, calcium, creatinine, total protein, aspartate aminotransferase (AST), alanine aminotransferase (ALT), urea, cholesterol, glucose, triglycerides, and C-reactive protein in the serum samples (Franzoni et al. 2026). For assay setup and validation, each biochemical and inflammatory parameter was evaluated using a within-subject approach, comparing post-immunization values to the corresponding baseline measurement obtained from the same animal prior to immunization. Biochemical analysis was performed on 10 animals, which were randomly divided into two groups: five animals from Group 1 (high-dose–single administration), whereas five wild boar were from Group 2 (low-dose–high-dose boost).

### Clinical signs and euthanasia

The animals were observed daily throughout the study to monitor their health status by a video surveillance system (Hikvision iVMS-4200, Hikvision®, Hangzhou, China), and visits by veterinarians. The evolution of the disease was expressed in terms of a quantitative clinical score (CS) specific for ASFV infection in wild boar previously described by Cadenas-Fernández et al. (2020). These signs included fever, behavior, body condition, skin alterations, ocular or nasal discharge, joint swelling, and respiratory, digestive, and neurological clinical signs. The degree of severity of each sign was measured from 0 to 4 (most severe). Fever was defined as a body temperature above 40 °C.

Animals that reached the pre-defined human endpoint (CS > 18) or showed any severe clinical signs (level 4) for more than two consecutive days were euthanized via intravenous injection of the T61® agent (Intervet, Spain) following anaesthesia by intramuscular injection of a combination of tiletamine–zolazepam (Zoletil 100 mg/mL, Virbac, France) and medetomidine (Medetor, Virbac, France) (Barasona et al. 2013).

### Tissue sampling and virological testing

A total of 20 tissue samples were collected from each animal, including lymph nodes (mandibular, renal, mediastinal, retropharyngeal, mesenteric, prescapular, gastrohepatic, inguinal lymph nodes), spleen, liver, lung, heart, kidney, brain, urinary bladder, intestine, diaphragm, bone marrow, and synovial membranes (Rodríguez-Bertos et al. 2020). The presence of ASFV DNA was tested employing the UPL qPCR technique (Fernandez-Pinero et al. 2013). Virus isolation was performed on positive tissue samples (Cq < 35) following standard operating procedures (SOPs) in porcine blood monocyte (PBM) cells, according to the recommendation of the European Union Reference Laboratory for ASF (http://asf-referencelab.info/asf/images/ficherosasf/PROTOCOLOS-EN/SOP-ASF-VI-1.pdf). To complement the qPCR data and verify the presence of the infectious virus in the host, blood and tissue samples were subjected to viral isolation. Samples were passed three times, and the plates were examined for haemadsorption over a period of 5 days.

### Data analysis

We first conducted an exploratory description of the main variables under study, including body temperature, clinical score values, antibody responses, ASFV viremia in blood (Cq values), and ASFV DNA presence in tissues. Mean values and ranges were estimated for each experimental group and sampling point, together with their 95% confidence intervals. The distribution of all independent variables was assessed using the Shapiro–Wilk test. Depending on normality, comparisons between groups or time points were made using either parametric or non-parametric methods. Specifically, differences between pre-immunization (day 0) and subsequent time points (12, 30, 35, 41, 48, and 61 dpi) were analyzed by ANOVA followed by Dunnett’s multiple comparison test when the data showed normal distribution, or by the Kruskal–Wallis test followed by Dunn's test in the case of non-parametric distributions.

Data obtained from the eGFP ELISA and pEP153R ELISA were statistically analyzed using frequency tables to calculate sensitivity and specificity, considering the INgezim® PPA Compac assay as a reference technique. Confidence intervals (95% CI) were estimated using the Clopper‒Pearson test for one proportion. To compare the two sensitivity percentages, a chi-square test was performed.

Group comparisons at single time points were further evaluated using the Mann–Whitney *U* test. All the statistical procedures were performed with SPSS v25 (IBM, Somar, NY, USA), R software v3.5.0, GraphPad Prism v10.01 (GraphPad Software Inc., La Jolla, CA, USA), and MedCalc® software, version 10.1.7.0. Differences were considered statistically significant at *p* < 0.05.

## Results

### Vaccination period

**Figure 2.**
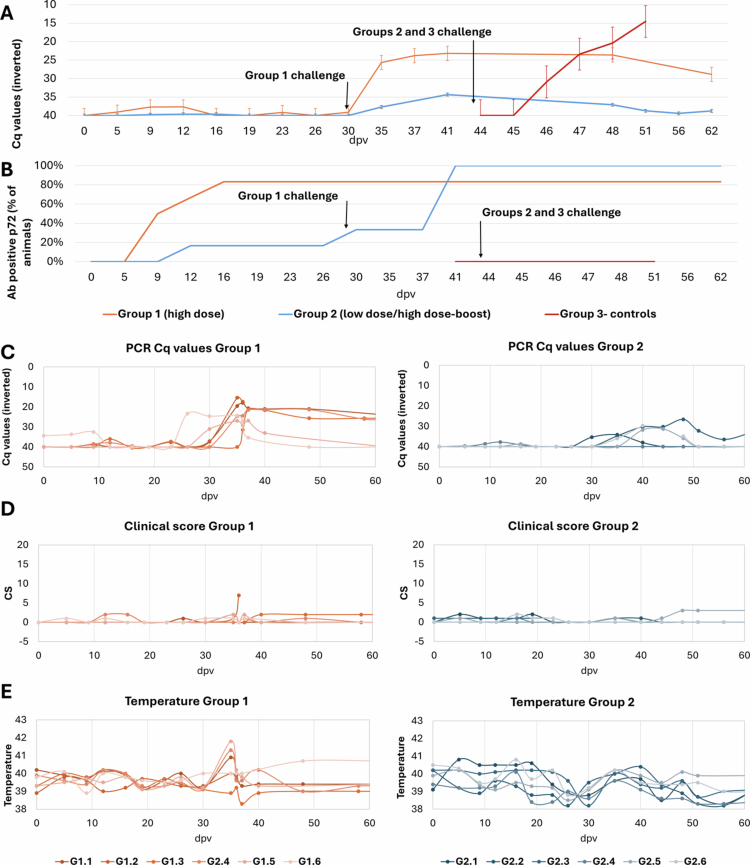
Dynamics of the Lv17/WB/Rie1-ΔCD vaccine candidate in wild boar. Panel (A) shows the averages of viremia, expressed in cycles of quantification (Cq) values of real-time PCR by group; (B) shows the percentage of wild boar with a positive antibody response (p72 ELISA) by group. Panels (C), (D), and (E) provide a view of individual viremia, clinical scores, and temperatures for Groups 1 and 2, respectively. All data are plotted against days post-vaccination (dpv). Group 1: oral vaccination with 10⁴ TCID₅₀ (high-dose–single administration) or 10² TCID₅₀ (low-dose–high-dose re-exposure administration).

During the vaccination period, five out of six animals orally vaccinated within Group 1 showed a positive antibody response, starting at 10 ± 3 dpv (Figure 2b; Supplementary Figure 1). After the prime vaccination, four out of six animals had a slight fever (40.12 °C ± 0.08 °C; CS = 0.67 ± 0.47) at 12 dpv, and this was observed on only one sampling day. These animals had sporadic viremia with high Cq values (Cq = 39.18 ± 1.74) (Figure 2a), except for one in which viremia was not detected, and corresponded to an animal that remained seronegative.

In Group 2, only one animal had detectable viremia during three consecutive sampling days (Cq = 38.52 ± 0.57), showing a slight fever (40.1 °C) and an ASFV-specific antibody response beginning at 12 dpv. None of the remaining five animals showed detectable antibodies during the primary vaccination phase. Following the second oral administration at 30 dpv, five out of the six animals exhibited viremia (Cq = 36.00 ± 3.82), accompanied by slight fever (40.1 °C ± 0.1 °C; CS = 0.57 ± 0.39), and all the animals seroconverted at 41 dpv. Throughout the vaccination period, no clinical signs other than mild hyperthermia were observed. All the animals from Group 2 were seropositive before IM challenge with the virulent ASFV Arm07 isolate. Statistical analysis revealed no significant differences between Groups 1 and 2 during the vaccination period, regarding viremia levels (Mann‒Whitney *U* = 23.5, *p* > 0.05) or clinical scores (Mann‒Whitney *U* = 53.5, *p* > 0.05).

### Challenge period

All successfully immunized animals (those showing an antibody response) survived the IM challenge with the highly virulent ASFV Arm07 isolate, with protective efficacies of 83.33% in Group 1 (high-dose single administration) and 100% in Group 2 (low-dose–high-dose boost). In contrast, all the animals in the control group developed severe ASF-compatible clinical signs and were euthanized at 6 and 7 days post-challenge (dpc), reaching a CS among 6 and 14.

During the challenge period, only mild signs were observed among the immunized animals in Group 1, characterized by a transient fever peak (40.5 °C ± 0.9 °C at 5 dpc/35 dpv), lethargy, and anorexia (CS = 1.38 ± 0.69). Moderate viremia was detected during this period (Cq = 25.62 ± 5.46) (Figure 2a). However, no fever or lethargy was observed in any animal from Group 2, and low viremia was detected in only two animals (Cq = 38.35 ± 3.55). The single non-immunized wild boar in Group 1 failed to survive the challenge, exhibiting severe clinical signs (CS = 14) and high viremia (Cq = 16.41 ± 1.0), and was euthanized at 6 dpc (36 dpv). Following the virulent challenge (D30–D62), the two-administration strategy in Group 2 provided significantly superior protection compared to the single-dose regime. Statistical analysis confirmed that Group 2 maintained significantly lower viral loads in the blood (Mann‒Whitney *U* = 4.0; *p* = 0.015) and a marked reduction in clinical signs (*U* = 22.5; *p* = 0.041) compared to Group 1.

### Inflammatory markers

**Figure 3.**
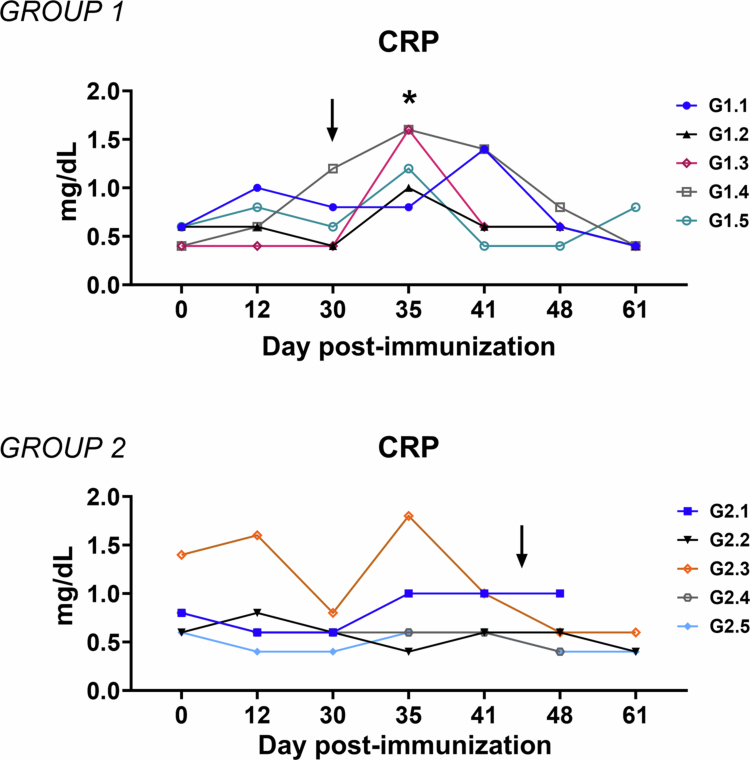
Kinetics of C-reactive protein levels in sera samples taken throughout the experiment. Wild boars were immunized with a high-dose single administration (10^4 ^TCID_50_) of Lv17/WB/Rie1-ΔCD (Group 1, *n* = 5) or low-dose–high-dose re-exposure of Lv17/WB/Rie1-ΔCD (10^2^ and 10^4^ of TCID_50_) (Group 2, *n* = 5). Wild boars were challenged with the ASFV Arm07 isolate at 30 dpv in Group 1 or at 44 dpv in Group 2. Sera samples were collected at different times during the *in vivo* experiment (days 0, 12, 30, 35, 41, 48, and 61). Changes in the C-reactive protein levels were monitored through ELISA. At each time point immunization, the values were compared compared to those pre-immunization (day 0); *p < 0.05.

In the present study, serum CRP levels were monitored throughout the experiment to evaluate systemic inflammation. Immunization with either one or two doses of the attenuated candidate vaccine Lv17/WB/Rie1-ΔCD did not induce any significant increase in CRP levels compared to baseline (Figure 3).

Upon challenge with Arm07, differences in CRP kinetics were observed between the experimental vaccinated groups. In Group 1 (high-dose single administration), a transient increase in CRP levels was observed at 5 dpc (35 dpi). In contrast, Group 2 (low-dose/high-dose boost) did not show a comparable increase at this time point. Among Group 1 animals, two individuals (G1.3 and G1.4) displayed the highest CRP values at 35 dpi. However, CRP levels in these animals returned to baseline at later time points, indicating that even a single dose of Lv17/WB/Rie1-ΔCD was sufficient to prevent sustained inflammation following ASFV challenge.

### Biochemical markers

To comprehensively assess the impact of immunization on animal health, a panel of 11 biochemical parameters was evaluated at multiple time points during the *in vivo* experiment.

**Figure 4.**
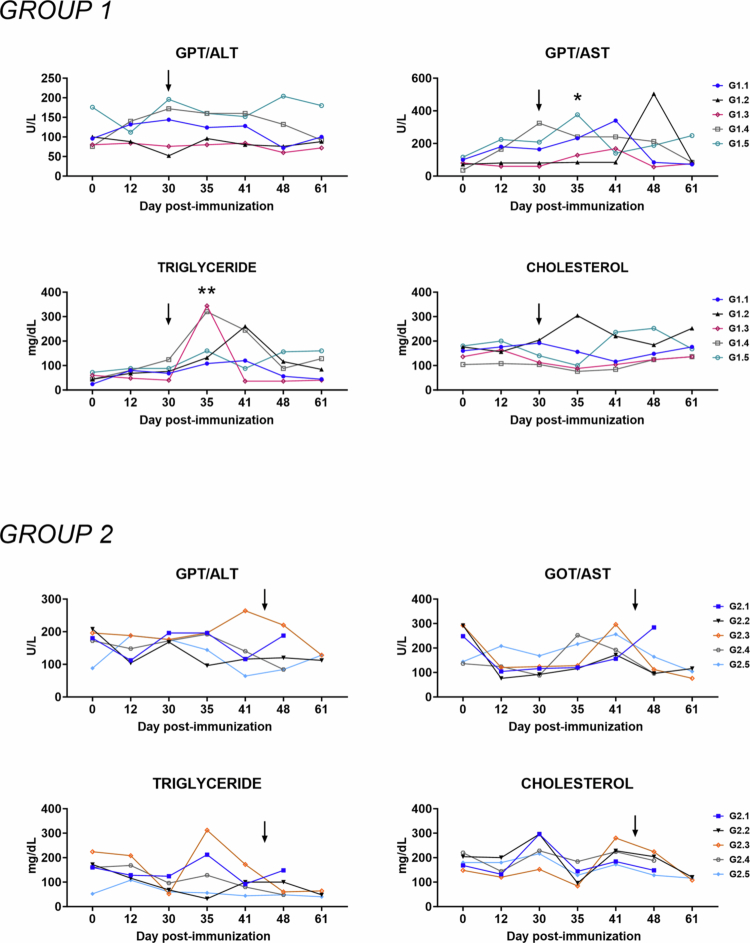
Kinetics of markers of hepatic function in sera samples taken throughout the experiment. Wild boars were immunized with a high-dose–single administration (10^4 ^TCID_50_) of Lv17/WB/Rie1-ΔCD (Group 1, *n* = 5) or low-dose–high-dose re-exposure of Lv17/WB/Rie1-ΔCD (10^2^ and 10^4^ of TCID_50_) (Group 2, *n* = 5). Wild boars were challenged with the ASFV Arm07 isolate at 30 dpv in Group 1 or at 44 dpv in Group 2. Sera samples were collected at different times during the *in vivo* experiment (days 0, 12, 30, 35, 41, 48, and 61), and then changes in the serum levels of GOT/AST, GPT/ALT, triglyceride, and cholesterol were subsequently monitored. At each immunization time point, values were compared with those recorded pre-immunization (day 0); *p < 0.05; **p < 0.01.

Serum levels of aspartate aminotransferase (AST/GOT), alanine aminotransferase (ALT/GPT), triglycerides, and cholesterol were measured as indicators of hepatic function (Figure 4). Immunization with either one or two doses of Lv17/WB/Rie1-ΔCD did not cause significant alterations in any of these markers compared to pre-immunization values, suggesting no hepatic toxicity associated with the candidate vaccine.

Following the ASF challenge, a transient increase in AST/GOT and triglyceride levels was observed in Group 1 but not in Group 2. In particular, individuals in G1.1, G1.4, and G1.5 in Group 1 showed increased ALT/GPT levels at 35 dpi, while G1.3 and G1.4 exhibited elevated triglycerides (Figure 4). These alterations returned to baseline at subsequent time points, further indicating that a single dose of the vaccine mitigated virus-induced liver dysfunction, although two doses provided superior protection.

**Figure 5.**
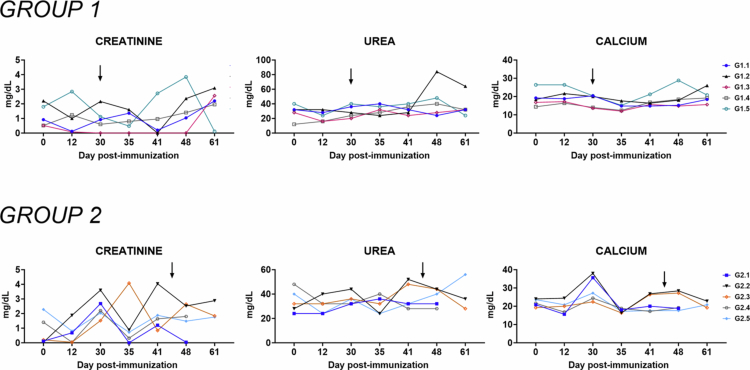
Kinetics of markers of kidney function in sera samples taken throughout the experiment. Wild boars were immunized with a high dose-single administration (10^4 ^TCID_50_) of Lv17/WB/Rie1-ΔCD (Group 1, *n* = 5), or a low-dose–high-dose re-exposure of Lv17/WB/Rie1-ΔCD (10^2^ and 10^4^ of TCID_50_) (Group 2, *n* = 5). Wild boars were challenged with the ASFV Arm07 isolate at 30 dpv in Group 1 or at 44 dpv in Group 2. Sera samples were collected at different times during the *in vivo* experiment (days 0, 12, 30, 35, 41, 48, and 61), and then, changes in the serum levels of creatinine, urea, and calcium were monitored. At each immunization time point, the values were compared with those recorded pre-immunization (day 0).

Renal function was assessed via the serum levels of creatinine and urea (Figure 5). No significant variations were observed in either biomarker following immunization with one or two doses of Lv17/WB/Rie1-ΔCD, suggesting the absence of nephrotoxicity. Moreover, no increases were detected post-challenge in either group, indicating that vaccinated animals were protected against ASFV-associated renal impairment.

**Figure 6.**
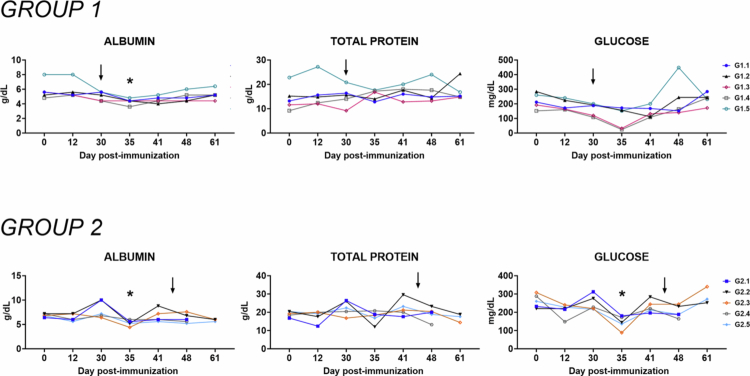
Kinetics of albumin, total protein, and glucose levels in sera samples taken throughout the experiment. Wild boars were immunized with a high dose-single administration (10^4 ^TCID_50_) of Lv17/WB/Rie1-ΔCD (Group 1, *n* = 5), or a low-dose–high-dose re-exposure of Lv17/WB/Rie1-ΔCD (10^2^ and 10^4^ TCID_50_) (Group 2, *n* = 5). Wild boars were challenged with the ASFV Arm07 isolate at 30 dpv in Group 1 or at 44 dpv in Group 2. Sera samples were collected at different times during the *in vivo* experiment (0, 12, 30, 35, 41, 48, and 61 dpv), and then changes in the serum levels of albumin, total protein, and glucose were monitored. At each immunization time point, the values were compared with those recorded pre-immunization (day 0).

Levels of serum albumin, total protein, and glucose were also measured throughout the study (Figure 6). No significant changes were observed following immunization or after ASFV challenge in either group. These findings are consistent with the absence of systemic metabolic disturbances and support the overall safety and protective efficacy of the candidate vaccine.

### Viral distribution in tissues

**Figure 7.**
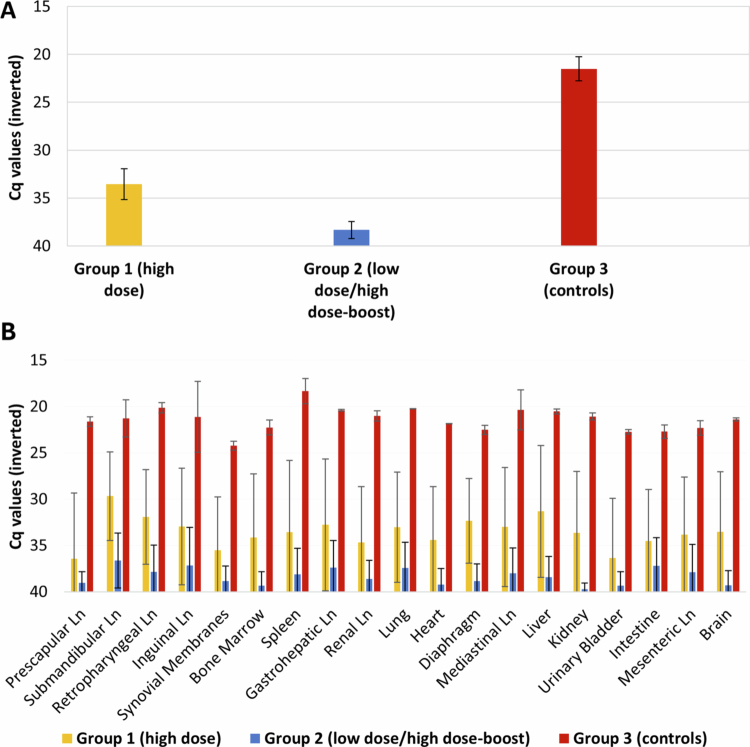
ASFV DNA levels in tissues. (A) ASFV DNA levels expressed as an average of Cq values from real-time PCR in sample tissues for wild boars orally vaccinated with a high-dose–single administration 10^4 ^TCID_50_ (Group 1), and low-dose–high-dose, re-exposure 10^2 ^TCID_50_/10^4 ^TCID_50_ (Group 2) of the Lv17/WB/Rie1-ΔCD isolate, and the control group infected with the Arm07 isolate. (B) ASFV DNA levels expressed as an average of Cq values from real-time PCR per sample tissue.

A total of 280 tissue samples were analyzed by qPCR. Group 1 presented 71.7% of positive tissues (an average of 14 tissues/animal), with Cq values of 33.66 ± 0.79, compared with 38.3% of positive tissues (an average of 8 tissues/animal) in the Group 2, with Cq values of 38.35 ± 0.96. Only one animal did not exhibit the presence of ASFV DNA in tissues, this animal was successfully immunized after prime vaccination with 10^2 ^TCID_50_ of Lv17/WB/Rie1-ΔCD isolate (Group 2). All the tissue samples (100%) from the control animals were positive for the detection of ASFV DNA, with an average of Cq values of 21.52 ± 1.84 (Figure 7 and Supplementary Figure 2). In a similar way, the one orally vaccinated animal from Group 1 that remained unprotected against challenge showed strongly positive PCR results (Cq = 21.31 ± 1.86) in all 20 tissues tested (Figure 7).

Virus isolation was performed on qPCR-positive tissues to assess the presence of infectious virus and determined its haemadsorption capacity. A total of 43 tissue samples (Cq < 35) were selected, including 23 remaining from Group 1 and 20 from Group 2.

In Group 1, ASFV was successfully isolated from 18 tissues. The vaccination isolate was detected in only two tissues—submandibular lymph nodes and the liver—of a single animal. The remaining 16 tissues yielded haemadsorbing virus, corresponding to the virulent Arm07 isolate. In Group 2, only five tissues tested positive for virus isolation. Two of these samples (gastrohepatic lymph node and lung) contained the non-haemadsorbing Lv17/WB/Rie1-ΔCD isolate, and three yielded the haemadsorbing Arm07 isolate.

### DIVA analysis

**Figure 8.**
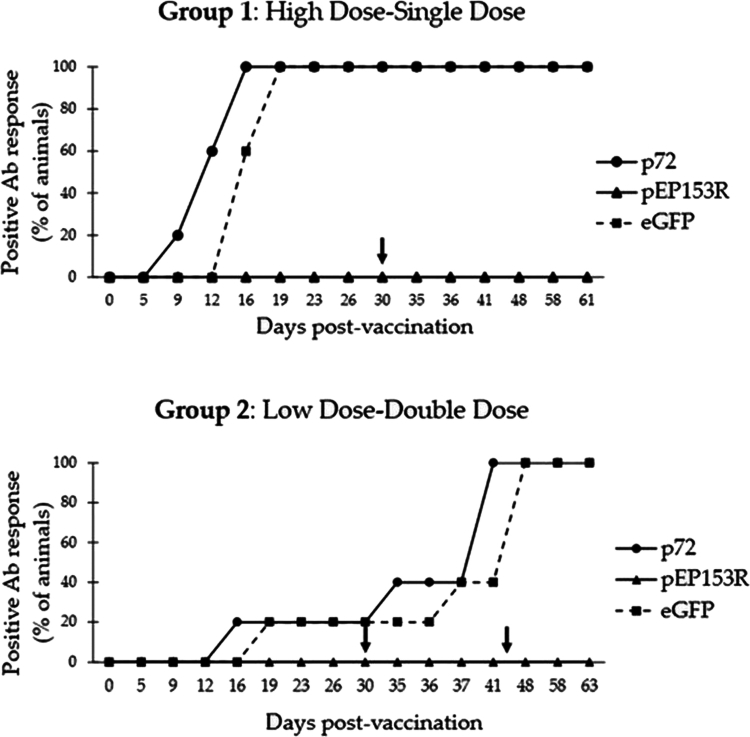
Serological DIVA response in Group 1 (above) and Group 2 (below). In each graph, the Y-axis represents the percentage of animals that tested positive by the INgezim® PPA Compac (p72), pEP153R ELISA (pEP153R), or eGFP ELISA (eGFP), while the X-axis indicates the days post-vaccination. The arrows mark the time point of challenge or second oral administration. Ab = antibody.

In the serological DIVA analysis, only animals that tested antibody positive for the p72 protein were considered. Five out of six animals from Group 1 (*n* = 57 samples) and all animals from Group 2 (*n* = 66 samples) fulfilled this criterion. All corresponding samples were re-tested using the same assay (INgezim® PPA Compac) to ensure consistency and to enable direct comparison across the three ELISAs that constitute the serological DIVA assay. To characterize the DIVA response, the resulting data were categorized according to the two immunization groups (Figure 8).

In Group 1, seroconversion against the p72 protein was detected at 13 ± 3 dpv, and 100% of the animals also tested positive by the eGFP ELISA. The mean seroconversion time based on the eGFP ELISA was 17 ± 2 dpv, following a trend similar to the antibody kinetics observed for the p72 protein. The eGFP ELISA demonstrated a sensitivity of 86.4% [95% CI: 73.3–93.6].

In Group 2, the animal that seroconverted prior to second oral administration tested positive by the INgezim® PPA Compac assay at 16 dpv and became positive by the eGFP ELISA 3 days later. The remaining animals, which seroconverted after the second vaccination, tested positive for both p72 and eGFP, with mean seroconversion times of 40 ± 3 dpv and 45 ± 6 dpv, respectively. In this group, the sensitivity of the eGFP ELISA was 83.3% [95% CI: 66.4–92.7], which was not statistically different from that observed in Group 1 (*p* > 0.05).

Regarding the third ELISA included in the DIVA test, the pEP153R ELISA, all animals from both groups tested negative up to the final sampling time point.

## Discussion

In recent years, ASF has emerged as a significant threat to the global pig industry, causing significant economic losses and disrupting trade flows (Pitts and Whitnall 2019). One of the primary epidemiological actors in the spread of ASF is the wild boar, which can act as a reservoir for the disease and facilitate the transmission of the virus to domestic pig populations (Jori et al. 2025). In this study, we provide data that may contribute to the development of vaccination strategies against ASF in wild boars and support ongoing efforts to control disease spread. The vaccine described here is based on a previously tested prototype that has been improved to increase its safety without affecting its effectiveness. The original prototype was based on the field Lv17/WB/Rie1 naturally live attenuated virus (Gallardo et al. 2019), which has proven to be highly effective in protecting against virulent ASF in both wild boar (Barasona et al. 2019) and domestic pigs (Gallardo et al. 2019). However, safety considerations were subsequently raised, particularly regarding the potential for residual virulence under certain conditions (Barasona et al. 2021). Notably, in that study, the single vaccinated animal that died did so in the context of a multifactorial event involving stress due to aggressive interactions within the pen, concomitant opportunistic infections, and the presence of the attenuated vaccine strain.

To address these concerns, an improved version of the vaccine has been developed that retains the same high level of protection against ASF while minimizing the risk of adverse effects. This development is crucial, especially considering that the global spread of ASF has led to a sharp decline in global swine production capacity, and the pig industry has been devastated (Zhang et al. 2023). Our results have shown that the vaccine is highly effective in protecting wild boars against ASF, with a high level of protection observed in vaccinated animals, mainly in Group 2. The safety of a vaccine is of utmost importance in ensuring its widespread use and effectiveness in preventing disease. The new vaccine candidate showed improved safety compared to previous studies conducted with the parental field strain (Barasona et al. 2021; Porras et al. 2025). This double-deletion mutant was previously characterized in domestic pigs by Gallardo et al. (2024), where it provided 100% protection against a virulent genotype II challenge. While some individuals showed transient and mild clinical reactions (subclinical viremia and occasional low-grade fever), the candidate proved to be significantly safer than its parental or single-deletion counterparts. Furthermore, as reported, the vaccine candidate maintains its attenuated phenotype even after five successive *in vivo* passages; although a slight residual virulence was observed in the last passage. In both models of vaccination tested in this study (high-dose–single administration and two-dose oral administration), no adverse effects were observed during the vaccination period. This favourable profile may be attributed to the dual gene deletion (EP402R/CD2v and EP153R) present in the vaccine candidate strain (Gallardo et al. 2024; Zhao et al. 2023).

The contribution of CD2v to virulence in genotype II ASFV isolates remains complex and context dependent. While deletion of CD2v alone from the Georgia strain does not significantly attenuate virulence in swine (Borca et al. 2020b), other studies have shown that modifications of CD2v in combination with additional gene deletions can markedly influence viral replication and protective efficacy (Gladue et al. 2020; Reis et al. 2025). These findings support the view that CD2v is not an independent determinant of attenuation in genotype II viruses but rather acts within a multifactorial virulence network. Although deletion of CD2v alone has not been shown to significantly attenuate highly virulent genotype II isolates, but in combination with EP153R, in an already attenuated genetic background, such as Lv17/WB/Rie1, may contribute to limiting viral persistence and immunomodulatory effects. Deletion of both EP402R/CD2v and EP153R may have reduced the likelihood of adverse effects observed following vaccination (Dixon et al. 2004; Gladue et al. 2020). These results indicate that this vaccine candidate has the potential to be an effective option for preventing infection. Following challenge, both vaccination regimens prevented lethal disease. Nevertheless, in this limited cohort, animals receiving a single 10⁴ TCID₅₀ oral dose tended to show higher post-challenge viral genome loads in the blood than animals that received two oral administrations. Importantly, the 10² TCID₅₀ first administration in the two-dose group did not consistently establish an immunization; thus, this schedule should not be interpreted as a classical prime–boost regimen. Rather, the data are most consistent with a re-exposure effect, whereby a second oral administration increases the probability of effective vaccine take across animals—an operationally relevant concept for multi-round baiting campaigns. Given the small group size, these observations should be considered preliminary and do not formally rank regimens. Despite the encouraging results observed in this study, a comprehensive safety evaluation remains necessary. While the findings suggest promising protective efficacy, dedicated safety studies are essential to fully assess the risk profile of the vaccine candidates under broader experimental and field conditions. These evaluations should involve larger numbers of animals and include potentially more susceptible categories, such as pregnant sows and animals of different age groups, to ensure a thorough characterization of the vaccine's safety profile across diverse scenarios.

Given the typical oronasal transmission route of ASF infection, it is plausible to hypothesize that the mucosal immune response might play a significant role in potentially limiting viral replication at the primary sites of entry, such as the tonsils and mucosa-associated lymphoid tissue (Xie et al. 2023). In the context of our orally administered vaccine, it might be speculated that the initiation of cellular immunity and the subsequent production of secretory IgA post-vaccination could be instrumental (Barroso-Arévalo et al. 2021). The evolution of this localized response might be a key factor in fostering a systemic protective response and the synthesis of virus-specific IgG antibodies. While these IgG antibodies are often considered as potential indicators of protection, their actual neutralizing activity remains uncertain. Based on current immunological knowledge, mucosal surfaces are considered primary barriers against a wide range of pathogens. Thus, vaccines targeting these surfaces might not only induce local immunity but could also stimulate systemic immune responses. Considering all these factors, it is plausible that the efficacy of the mucosal response could be augmented through booster immunization. Notably, one animal in the re-exposure group showed evidence of vaccine take after the 10² TCID₅₀ oral prime. While this is a low dose, it is compatible with the biology of mucosal immunization, in which early events are highly localized and can be stochastic. Antigen uptake and immune priming can occur within organized mucosa-associated lymphoid tissue (MALT) in the oropharyngeal region, including tonsillar structures, where sampling by antigen-presenting cells and limited local replication may be sufficient to initiate an adaptive response once a critical threshold is reached. In contrast, the same nominal dose may fail in other animals if the effective mucosal exposure is shorter or if local innate defences and barrier factors prevent productive infection. Thus, this observation suggests that 10² TCID₅₀ may occasionally be sufficient for oral uptake, but our group-level data indicate that it is below a dose that reliably immunizes all animals under the conditions tested.

For the animals in Group 2, which received a high-dose second administration, the progression of infection post-challenge with the virulent Arm07 isolate differed from that of the single-dose group. Notably, the revaccinated animals did not exhibit clinical signs such as fever or lethargy after challenge, which were evident in the single-dose group. Additionally, the spread of the challenge strain within the tissues was significantly reduced in the revaccinated wild boar, indicating a more robust and highly protective immune response that effectively controlled the replication of the virulent strain.

The prime–re-exposure strategy evaluated in Group 2 was intended to mimic a plausible field scenario in which wild boar may encounter vaccine baits on more than one occasion during repeated distribution campaigns. Although the second administration was part of the planned design, the decision to administer a higher dose at the second oral administration was primarily motivated by the limited antibody response observed after the initial low-dose immunization, allowing us to further explore the immunogenic potential of the construct under oral delivery conditions.

These findings suggest that several immunizations combined with higher doses may represent a more effective vaccination strategy for oral administration. It is important to take into account the inherent lack of control over bait uptake in the field, which makes it necessary to evaluate the effects of multiple exposures. In this regard, the safety profile observed after a 10^4 ^TCID_50_ exposure is particularly relevant, as it ensures that individuals ingesting multiple baits are not at risk of developing vaccine-derived pathology. Furthermore, these results support the use of pulsed baiting campaigns, a strategy previously validated for the control of classical swine fever and tuberculosis (Díez-Delgado et al. 2018; Rossi et al. 2015). These findings may also contribute to the optimization of bait-based strategies currently being investigated for ASF in wild boar populations (Pachuri et al. 2024; Relimpio et al. 2024). Future studies should investigate the optimal vaccine dosage and the need for repeated doses to ensure sustained immunity, as well as the underlying mechanisms of the vaccine that confer protection.

Regarding the inflammatory markers analyzed, several interesting results were observed. CRP is a major acute phase protein (APP) and is widely used as a marker for inflammation (Petersen et al. 2004). Previous studies reported that infection with virulent ASFV isolates resulted in inflammation, identified by high CRP concentration in sera (Sánchez-Cordón et al. 2007; Walczak et al. 2021; Franzoni et al. 2023). Our data revealed that immunization with either one or two doses of Lv17/WB/Rie1-ΔCD did not trigger an increase in CRP, suggesting that this candidate vaccine was highly tolerated by wild boars. After challenge, a transient increase in CRP serum levels was observed in animals receiving only a single dose of Lv17/WB/Rie1-ΔCD but not in those receiving a double immunization. Consistent with the virological results, the administration method for Group 2 showed a tendency toward lower acute-phase/inflammatory markers after challenge; however, because the first low-dose exposure did not reliably immunize most animals, these findings are better interpreted as potentially reflecting more consistent vaccine take after repeated oral exposure rather than a specific booster-mediated immunomodulatory effect. Importantly, we acknowledge that the absence of longitudinal biochemical monitoring in the intramuscularly challenged control group precludes direct statistical comparison of CRP responses between vaccinated and non-vaccinated animals. This represents a limitation of the present study and should be considered when interpreting the magnitude of the observed effects.

To provide a biological context, our findings were interpreted in light of previously reported biochemical responses in wild boars experimentally infected with the virulent genotype II ASFV (Franzoni et al. 2026). In this framework, the transient and self-resolving CRP changes observed in vaccinated animals contrast with the marked and sustained inflammatory responses typically described in acute virulent infections (Franzoni et al. 2026), providing contextual support for the interpretation of our findings. In Group 1, CRP serum levels returned to baseline levels at a later time post-challenge, indicating that even a single dose of the candidate vaccine was associated with control of systemic inflammation after challenge with the virulent ASFV isolate.

Four serum markers were monitored to evaluate liver functions: AST and ALP to monitor hepatocyte health and function, triglycerides and cholesterol to assess liver's metabolic capacity (Lee et al. 2012; Franzoni et al. 2026). Previous studies reported that virulent genotype II isolates (Pol18_28298_O111) triggered an increase in AST and a decrease in ALT serum levels as early as 2 days post-infection (Walczack et al. 2021). We observed that immunization with either candidate vaccine did not result in changes in the serum levels of AST, ALT, or other markers associated with liver damage or impairment (triglyceride and cholesterol levels) in any of the tested animals, indicating that the deleted vaccine did not trigger hepatic damage. We observed differences between groups in response to challenge. We observed a transient increase in both AST and triglyceride serum levels after challenge in Group 1 but not in Group 2. This suggests that a double dose of immunization provided a stronger protection against challenge with virulent ASFV. Nevertheless, the serum levels of both parameters returned to baseline levels at a later time post-challenge, indicating that even a single dose of the candidate vaccine strongly reduced liver damage caused by ASFV in the tested animals.

Markers of renal impairments, such as urea, creatine, and the electrolyte calcium, were next investigated (Lyman 1986). Previous studies showed that infection with virulent genotype II isolates (Pol18_28298_O111) triggered an increase of urea, and creatine levels in naive pigs at 2 days post-infection (Walczak et al. 2021). Immunization with either candidate vaccines did not trigger increased serum levels of these markers, highlighting the safety of that marker vaccine. In addition, no increase was observed after challenge with Arm07, showing that even a single dose of the candidate vaccine conferred solid protection and prevented ASFV-related renal damage.

Finally, the serum levels of albumin, total protein, and glucose were monitored. Infection with Arm07 resulted in increased serum levels of albumin and total protein in domestic pigs, maybe due to dehydration caused by the high fever, which triggers a decrease in the plasmatic volume with a relative increase of albumin concentration. Infection with Arm07 triggered also increased serum levels of glucose in domestic pigs (Franzoni et al. 2026), likely due to the release of stress hormones, which are released by the host to overcome the infection. These changes were not observed in this study, either after immunization or after challenge. These data highlighted the safety of this candidate vaccine in wild boars and also suggested that immunization provided protection against challenge with virulent ASFV.

In the context of ASF control, oral vaccination using baits formulations represents the most promising strategy (Díez-Delgado et al. 2018; Rossi et al. 2015; Relimpio et al. 2024; Pachauri et al. 2024). This method is considered both safe and effective, as it minimizes the need for direct human intervention and reduces the risk of virus dissemination during animal handling. However, it presents a major challenge regarding the monitoring and traceability of vaccinated individuals (Beltrán-Beck et al. 2014), potentially comprising the ASF-free status. Moreover, viremia in vaccinated wild boar tends to be transient (Porras et al. 2024), which limits the sensitivity of direct virus detection methods (Relimpio et al. 2025). Therefore, reliable serological differentiation between vaccinated and infected animals becomes essential for effective surveillance. To address this key aspect, we used a genetic modification that confers DIVA capabilities (Pasick 2004). This is achieved by deleting a gene encoding a viral antigenic protein and inserting a specific antigenic marker present exclusively in the vaccine strain and absent from circulating field isolates. Both the negative and positive markers, along with a control antigen such as the p72 protein, can be detected using a simple and cost-effective ELISA-based test.

Considering this, we further evaluated the assay previously described by González-García et al. (2025) as a DIVA diagnostic tool. As shown by the results presented here (Figure 8), vaccinated animals were identified based on a negative antibody response against the negative marker of the vaccine, pEP153R, in conjunction with a positive response against both the control protein (p72) and the inserted positive marker (eGFP). This protein provides a measurable proof-of-concept for differentiating vaccinated animals from infected animals. In this construct, GFP was incorporated as a model positive marker to demonstrate the feasibility of a ‘positive DIVA’ approach under experimental conditions. We note that GFP is used here as a proof-of-concept and may not represent the most suitable marker for a final field-deployable product, which could instead rely on alternative marker strategies more aligned with regulatory and operational requirements. Furthermore, the selection of *EP153R* gene for deletion follows an established lineage of research aimed at optimizing the safety and diagnostic capabilities of ASFV vaccines. Previous studies have shown that the Lv17/WB/Rie1-ΔCD construct maintains robust protection while exhibiting a superior safety profile compared to its parental predecessor (Gallardo et al. 2024) (Supplementary Table 1). On the other hand, the absence of detectable antibodies against pEP153R, even after challenge, suggests that the viral replication of the Arm07 virulent strain is limited to trigger a detectable antibody response against this marker until the last sample analyzed. In contrast, the antibody response against eGFP did not differ between the two immunization groups, indicating that the immunization strategy seems to not influence the response to this marker (Gonzalez-García et al. 2025).

Therefore, the vaccine prototype with DIVA properties presented in this study is a significant breakthrough in the fight against this disease, particularly in the context of wild boar vaccination programs. Its ability to differentiate between infected and vaccinated animals is critical for ensuring that disease-free status is maintained and that the spread of the disease is accurately tracked. By using this vaccine, we can take a major step towards controlling this disease and protecting both wild and domestic animals from its devastating effects. In addition, these outcomes show that the vaccine is safe and effective, providing a promising avenue for controlling the spread of ASF in wild boar populations and protecting the global pig industry from this devastating disease. However, further research is required to address several aspects that will enrich the perspective of wild boar vaccination with this vaccine prototype. First, the deletion virus used as the vaccine has been grown and produced using porcine alveolar macrophages. Primary cell lines often pose limitations in scalability, consistency, and long-term sustainability, which are crucial for large-scale vaccine production (Meloni et al. 2022). Furthermore, to truly ascertain the vaccine's efficacy and safety, it is essential to test it on a broader spectrum of animals, ensuring its robustness in diverse scenarios. Another pivotal aspect that warrants exploration is the longevity of the immunity conferred by the vaccine. As time progresses, understanding the duration and persistence of the immune response becomes paramount in order to establish timelines and vaccination programs in both domestic and wild populations. In conclusion, while the strides made are commendable and hold immense promise for the future, the journey ahead is intricate, demanding a blend of meticulous research, innovation, and collaboration.

## Acknowledgements

We are deeply grateful to the VACDIVA consortium for providing the ASFV isolates used in this study, with special thanks to Carmina Gallardo, Marisa Arias, Antonio Rodríguez-Bertos, José M. Sánchez-Vizcaíno, Erwin van den Born, and Zoltán Zádori. We also sincerely thank Rocío Sánchez and Débora López for their invaluable technical support and assistance throughout the experimental work, as well as the entire SCB-VISAVET team for their essential contributions.

We further acknowledge the crucial support of the ANEC (Animal Facility Unit, VISAVET) team for their assistance with animal handling and experimental procedures, and the SAP (Pathology and Forensic Veterinary Unit, VISAVET) group for their expert work in conducting necropsies.

## Supplementary Material

SUPPLEMENTARY MATERIAL.docx SUPPLEMENTARY MATERIAL.docx

## Data Availability

The datasets generated and analyzed during the current study are included in the paper; further inquiries can be directed to the corresponding author.

## References

[cit0001] Alonso C et al. 2018. ICTV virus taxonomy profile: asfarviridae. J Gen Virol. 99(5):613–614. 10.1099/jgv.0.001049 29565243 PMC12662184

[cit0002] Arias M, Sánchez-Vizcaíno JM. 2012. African swine fever. In: Zimmerman J, Karriker LA, Ramirez A, Schwartz KJ, Stevenson GW, editors. Diseases of swine. 10th ed. Wiley. p 396–404.

[cit0003] Arias M, Sánchez-Vizcaíno JM, Morilla A, Yoon KJ, Zimmerman JJ. 2002. African swine fever. In: Trends in emerging viral infections of swine. Morilla A, Yoon KJ, Zimmerman JJ. Blackwell Publishing Company: Iowa State. p 119–124. 10.1002/9780470376812

[cit0004] Barasona JA et al. 2019. First oral vaccination of Eurasian wild boar against African swine fever virus genotype II. Front Vet Sci. 6:137. 10.3389/fvets.2019.00137 31106218 PMC6498142

[cit0005] Barasona JA et al. 2021. Safety of African swine fever vaccine candidate Lv17/WB/Rie1 in wild boar: overdose and repeated doses. Front Immunol. 12:761753. 10.3389/fimmu.2021.761753 34917082 PMC8669561

[cit0006] Barasona JA, López-Olvera JR, Beltrán-Beck B, Gortázar C, Vicente J. 2013. Trap-effectiveness and response to tiletamine-zolazepam and medetomidine anaesthesia in Eurasian wild boar captured with cage and corral traps. BMC Vet Res. 9(1):107. 10.1186/1746-6148-9-107 23702232 PMC3665459

[cit0007] Barroso-Arévalo S, Barasona JA, Cadenas-Fernández E, Sánchez-Vizcaíno JM. 2021. The role of interleukine-10 and interferon-γ as potential markers of the evolution of African swine fever virus infection in wild boar. Pathogens. 10(6):757. 10.3390/pathogens10060757 34203976 PMC8232672

[cit0008] Beltrán-Beck B et al. 2014. Assessment of an oral *Mycobacterium bovis* BCG vaccine and an inactivated *M. bovis* preparation for wild boar in terms of adverse reactions, vaccine strain survival, and uptake by nontarget species. Clin Vaccine Immunol. 21(1):12–20. 10.1128/CVI.00488-13 24173022 PMC3910919

[cit0009] Borca MV et al. 2020a. Development of a highly effective African swine fever virus vaccine by deletion of the I177L gene results in sterile immunity against the current epidemic Eurasia strain. J Virol. 94(7):10–1128. 10.1128/JVI.02017-19 PMC7081903 31969432

[cit0010] Borca MV et al. 2020b. Deletion of CD2-like gene from the genome of African swine fever virus strain Georgia does not attenuate virulence in swine. Sci Rep. 10(1):494. 10.1038/s41598-020-57455-3 31949276 PMC6965178

[cit0011] van den Born E et al. 2025. African swine fever virus vaccine strain Asfv-G-∆I177l reverts to virulence and negatively affects reproductive performance. NPJ Vaccines. 10(1):46. 10.1038/s41541-025-01099-9 40050309 PMC11885574

[cit0012] Bosch-Camós L, López E, Rodriguez F. 2020. African swine fever vaccines: a promising work still in progress. Porcine Health Manag. 6:1–14. 10.1186/s40813-020-00154-2 32626597 PMC7329361

[cit0013] Cadenas-Fernández E et al. 2020. Adenovirus-vectored African swine fever virus antigens cocktail is not protective against virulent Arm07 isolate in Eurasian wild boar. Pathogens. 9(3):171. 10.3390/pathogens9030171 32121082 PMC7157622

[cit0014] Cadenas-Fernández E et al. 2021. High doses of inactivated African swine fever virus are safe, but do not confer protection against a virulent challenge. Vaccines. 9(3):242. 10.3390/vaccines9030242 33802021 PMC7999564

[cit0015] Cadenas-Fernández E et al. 2024. Challenging boundaries: is cross-protection evaluation necessary for African swine fever vaccine development? A case of oral vaccination in wild boar. Front Immunol. 15:1388812. 10.3389/fimmu.2024.1388812 39411716 PMC11473374

[cit0016] Danzetta ML, Marenzoni ML, Iannetti S, Tizzani P, Calistri P, Feliziani F. 2020. African swine fever: lessons to learn from past eradication experiences. A systematic review. Front Vet Sci. 7:296. 10.3389/fvets.2020.00296 32582778 PMC7296109

[cit0017] Diez-Delgado I et al. 2018. Impact of piglet oral vaccination against tuberculosis in endemic free-ranging wild boar populations. Prev Vet Med. 155:11–20. 10.1016/j.prevetmed.2018.04.002 29786520

[cit0018] Dixon LK et al. 2004. African swine fever virus proteins involved in evading host defence systems. Vet Immunol Immunopathol. 100(3-4):117–134. 10.1016/j.vetimm.2004.04.002 15207450

[cit0019] European Food Safety Authority (EFSA), Ståhl K, Boklund AE, Podgórski T, Vergne T, Abrahantes JC, Mur L. 2024. Epidemiological analysis of African swine fever in the european union during 2023. EFSA J. 22(5):e8809. 10.2903/j.efsa.2024.8809 38756349 PMC11096997

[cit0020] Fernández‐Pinero J et al. 2013. Molecular diagnosis of African swine fever by a new real‐time PCR using universal probe library. Transbound Emerg Dis. 60(1):48–58. 10.1111/j.1865-1682.2012.01317.x 22394449

[cit0021] Food and Agriculture Organization of the United Nations F. 2025.African swine fever (ASF) situation update in Asia & Pacific. https://www.fao.org/animal-health/situation-updates/asf-in-asia-pacific/en

[cit0022] Franzoni G et al. 2023. Evaluation of haematological and immunological parameters of the ASFV Lv17/WB/Rie1 strain and its derived mutant Lv17/WB/Rie1/d110-11L against ASFV challenge infection in domestic pigs. Vaccines. 11:1277. 10.3390/vaccines11071277 37515092 PMC10383595

[cit0023] Franzoni G et al. 2026. Dynamics of leukocyte populations, immune-regulatory cytokines, and biochemical parameters in wild boar and domestic pigs experimentally infected with a virulent African swine fever virus genotype II strain. Front Immunol. 17:1751646. 10.3389/fimmu.2026.1751646 PMC13054600 41953012

[cit0024] Gallardo C et al. 2019. Attenuated and non‐haemadsorbing (non‐HAD) genotype II African swine fever virus (ASFV) isolated in Europe, Latvia 2017. Transbound Emerg Dis. 66(3):1399–1404. 10.1111/tbed.13132 30667598

[cit0025] Gallardo C et al. 2024. Double Deletion of EP402R and EP153R in the Attenuated Lv17/WB/Rie1 African Swine Fever Virus (ASFV) Enhances Safety, Provides DIVA Compatibility, and Confers Complete Protection Against a Genotype II Virulent Strain. Vaccines. 12:1406 1406. 10.3390/vaccines12121406 39772067 PMC11680264

[cit0026] Gladue DP et al. 2020. Deletion of CD2-like (CD2v) and C-type lectin-like (EP153R) genes from African swine fever virus Georgia-∆ 9GL abrogates its effectiveness as an experimental vaccine. Viruses. 12(10):1185. 10.3390/v12101185 33092057 PMC7590024

[cit0027] González-García G et al. 2025. A novel prototype African swine fever virus DIVA (Differentiation Between Infected and Vaccinated Animals) serological assay based on the detection of antibodies against the pEP153R, eGFP, and p72 proteins. Vaccines. 13(3):211. 10.3390/vaccines13030211 40266072 PMC11945662

[cit0028] Gortazar C, Diez-Delgado I, Barasona JA, Vicente J, De La Fuente J, Boadella M. 2015. The wild side of disease control at the wildlife-livestock-human interface: a review. Front Vet Sci. 1:27. 10.3389/fvets.2014.00027 26664926 PMC4668863

[cit0029] Guinat C et al. 2016. Transmission routes of African swine fever virus to domestic pigs: current knowledge and future research directions. Vet Rec. 178(11):262–267. 10.1136/vr.103593 26966305 PMC4819659

[cit0030] Jori F, Burnichon EP, Casal J, Barasona JA. 2025. Wild boar trade and African swine fever risk of introduction into new territories: a quantitative release assessment with retrospective data of wild boar shipments to France and Spain (2010–2017). One Health. 21:101185. 10.1016/j.onehlt.2025.101185 41036082 PMC12482638

[cit0031] Lee TH, Kim WR, Poterucha JJ. 2012. Evaluation of elevated liver enzymes. Clin Liver Dis. 16(2):183–198. 10.1016/j.cld.2012.03.006 22541694 PMC7110573

[cit0032] Lyman JL. 1986. Blood urea nitrogen and creatinine. Emerg Med Clin North Am. 4:223–233. 10.1016/S0733-8627(20)30997-4 3516645

[cit0033] Meloni D, Franzoni G, Oggiano A. 2022. Cell lines for the development of African swine fever virus vaccine candidates: an update. Vaccines. 10(5):707. 10.3390/vaccines10050707 35632463 PMC9144233

[cit0034] Nguyen TC et al. 2025. An African swine fever vaccine-like variant with multiple gene deletions caused reproductive failure in a Vietnamese breeding herd. Sci Rep. 15:14919. 10.1038/s41598-025-95641-3 40295549 PMC12037777

[cit0035] Olesen AS et al. 2020. Potential routes for indirect transmission of African swine fever virus into domestic pig herds. Transbound Emerg Dis. 67(4):1472–1484. 10.1111/tbed.13538 32150785

[cit0036] Pachauri R, Martínez-Guijosa J, Ferreras-Colino E, Ferreres J, Relimpio D. 2024. Optimizing the baiting strategy for oral vaccine delivery to wild boar. Eur J Wildl Res. 70:18. 10.1007/s10344-024-01771-w

[cit0037] Pasick J. 2004. Application of DIVA vaccines and their companion diagnostic tests to foreign animal disease eradication. Anim Health Res Rev. 5(2):257–262. 10.1079/ahr200479 15984335

[cit0038] Pepin KM, Golnar AJ, Abdo Z, Podgórski T. 2020. Ecological drivers of African swine fever virus persistence in wild boar populations: insight for control. Ecol Evol. 10(6):2846–2859. 10.1002/ece3.6100 32211160 PMC7083705

[cit0039] Pérez-Núñez D, Castillo-Rosa E, Vigara-Astillero G, García-Belmonte R, Gallardo C, Revilla Y. 2020. Identification and isolation of two different subpopulations within African swine fever virus Arm/07 stock. Vaccines. 8(4):625. 10.3390/vaccines8040625 33113838 PMC7712101

[cit0040] Petersen HH, Nielsen JP, Heegaard PM. 2004. Application of acute phase protein measurements in veterinary clinical chemistry. Vet Res. 35:163–187. 10.1051/vetres:2004002 15099494

[cit0041] Petrini S et al. 2023. The production of recombinant African swine fever virus Lv17/WB/Rie1 strains and their in vitro and in vivo characterizations. Vaccines. 11:1860. 10.3390/vaccines11121860 38140263 PMC10748256

[cit0042] Pitts N, Whitnall T. 2019. Impact of African swine fever on global markets. Agric Commod. 9(3):52–54.

[cit0043] Podgórski T, Śmietanka K. 2018. Do wild boar movements drive the spread of African swine fever? Transbound Emerg Dis. 65(6):1588–1596. 10.1111/tbed.12910 29799177

[cit0044] Porras N et al. 2025. Viral distribution of wild boar exposed to low (vaccine candidate) and high virulence African swine fever virus isolates: immunohistochemical characterization. Transbound Emerg Dis. 2025(1):4258247. 10.1155/tbed/4258247 41395245 PMC12697812

[cit0045] Porras N, Sánchez-Vizcaíno JM, Barasona JÁ, Gómez-Buendía A, Cadenas-Fernández E, Rodríguez-Bertos A. 2024. Histopathologic evaluation system of African swine fever in wild boar infected with high (Arm07) and low virulence (Lv17/WB/Riel) isolates. Vet Pathol. 61(6):928–942. 10.1177/03009858241266944 39078034

[cit0046] Reis AL et al. 2025. From structure prediction to function: defining the domain on the African swine fever virus CD2v protein required for binding to erythrocytes. mBio. 16(2):494. 10.1038/s41598-020-57455-3 PMC11796414 39688401

[cit0047] Relimpio D et al. 2024. Improved stability and specificity of baits for oral administration of substances to wild boar. Prev Vet Med. 229:106241. 10.1016/j.prevetmed.2024.106241 38878496

[cit0048] Relimpio D, Kosowska A, Barroso-Arévalo S, De Antonio-Gómez D, Gortázar C, Barasona JA. 2025. Oral fluid collection in wild boar: a field protocol. Vet J. 312:106362. 10.1016/j.tvjl.2025.106362 40273977

[cit0049] Rodríguez-Bertos A et al. 2020. Clinical course and gross pathological findings in wild boar infected with a highly virulent strain of African swine fever virus genotype II. Pathogens. 9(9):688. 10.3390/pathogens9090688 32842614 PMC7559345

[cit0050] Rossi S et al. 2015. Controlling of CSFV in european wild boar using oral vaccination: a review. Front Microbiol. 6:163928. 10.3389/fmicb.2015.01141 PMC4615961 26557109

[cit0051] Sánchez-Cordón PJ et al. 2007. Serum concentrations of C-reactive protein, serum amyloid A, and haptoglobin in pigs inoculated with African swine fever or classical swine fever viruses. Am J Vet Res. 68:772–777. 10.2460/ajvr.68.7.772 17605613

[cit0052] Sánchez-Vizcaíno JM, Mur L, Gomez-Villamandos JC, Carrasco L. 2015. An update on the epidemiology and pathology of African swine fever. J Comp Pathol. 152(1):9–21. 10.1016/j.jcpa.2014.09.003 25443146

[cit0053] Schäfer A, Franzoni G, Netherton CL, Hartmann L, Blome S, Blohm U. 2022. Adaptive cellular immunity against African swine fever virus infections. Pathogens. 11(2):274. 10.3390/pathogens11020274 35215216 PMC8878497

[cit0054] Schambow RA, Carrasquillo N, Kreindel S, Perez AM. 2025. An update on active and passive surveillance for African swine fever in the Dominican Republic. Sci Rep. 15(1):2244. 10.1038/s41598-025-86690-9 39833369 PMC11747335

[cit0055] Takamatsu HH et al. 2013. Cellular immunity in ASFV responses. Virus Res. 173(1):110–121. 10.1016/j.virusres.2012.11.009 23201582

[cit0056] Tran XH et al. 2022. African swine fever virus vaccine candidate ASFV‐G‐δI177L efficiently protects european and native pig breeds against circulating Vietnamese field strain. Transbound Emerg Dis. 69(4):e497–e504. 10.1111/tbed.14329 34582622

[cit0057] Urbano AC, Ferreira F. 2022. African swine fever control and prevention: an update on vaccine development. Emerg Microbes Infect. 11(1):2021–2033. 10.1080/22221751.2022.2108342 35912875 PMC9423837

[cit0058] Vu HL, McVey DS. 2024. Recent progress on gene-deleted live-attenuated African swine fever virus vaccines. NPJ Vaccines. 9(1):1–10. 10.1038/s41541-024-00845-9 38480758 PMC10937926

[cit0059] Walczak M et al. 2021. Blood counts, biochemical parameters, inflammatory, and immune responses in pigs infected experimentally with the African swine fever virus isolate Pol18_28298_O111. Viruses. 13:521. 10.3390/v13030521 33810057 PMC8004642

[cit0060] Xie Q et al. 2023. Dynamics of serological and mucosal antibody responses against African swine fever viruses in experimentally infected pigs. Transbound Emerg Dis. 2023:9959847. 10.1155/2023/9959847 40303747 PMC12017000

[cit0061] Zhang H et al. 2023. Vaccines for African swine fever: an update. Front Microbiol. 14:1139494. PMID: 37180260; PMCID: PMC10173882. 10.3389/fmicb.2023.1139494 37180260 PMC10173882

[cit0062] Zhao D et al. 2023. Highly lethal genotype I and II recombinant African swine fever viruses detected in pigs. Nat Commun. 14:3096. 10.1038/s41467-023-38868-w 37248233 PMC10226439

