# Supplementary material for: A dual-gene-deleted ASFV Lv17/WB/Rie1-ΔCD candidate administered orally to wild boar confers DIVA-compatible protection against virulent challenge
Source: Vet Q. 2026 Mar 26;46(1):2649573. doi: 10.1080/01652176.2026.2649573 (PMC13023007; doi:10.1080/01652176.2026.2649573)
Supplement: SUPPLEMENTARY MATERIAL.docx [file TVEQ_A_2649573_SM5220.docx]

**SUPPLEMENTARY MATERIAL**

**Supplementary Table 1**: Evolutionary characterization and optimization of the Lv17/WB/Rie1-based ASFV vaccine candidates.

| **Strain** | **Genetic modification** | **Host** | **Route** | **Dose** | **Protection** | **Safety** | **Reference** |
| --- | --- | --- | --- | --- | --- | --- | --- |
| Lv17/WB/Rie1 | None. Natural attenuation. Genotype II | DP | IM | 10 TCID_50_ | 100% | Transient fever and joint swelling | Gallardo et al., 2019 |
|  |  | WB | Oral | 10^3^ TCID_50_ double dose | 100% | Transient fever and lethargy | Barasona et al., 2021 |
|  |  |  |  | 10^4^ TCID_50_ double dose | 60% | Transient fever and lethargy. One WB high fever and succumbed during the vaccination period |  |
| Lv17/WB/Rie1-ΔEP153R | Single Deletion (pEP153R) | DP | IM | 10^2^ TCID_50_ | 66,6% | Transient clinical signs in most of the animals. Two DP developed severe signs and were euthanized | Gallardo et al., 2024 |
| Lv17/WB/Rie1-ΔCD | Double Deletion (ΔEP402R/ ΔEP153R) | DP | IM | 10^2^ TCID_50_ | 83,33% | Improved safety, mild subclinical signs |  |
|  | Double Deletion (ΔEP402R/ ΔEP153R) | WB | Oral | Double dose  10^2^ TCID_50_ + 10^4^ TCID_50_ | 100% | Slight and transient fever | This study |
|  |  |  |  | Single dose 10^4^ TCID_50_ | 83,33% | Slight and transient fever |  |

**
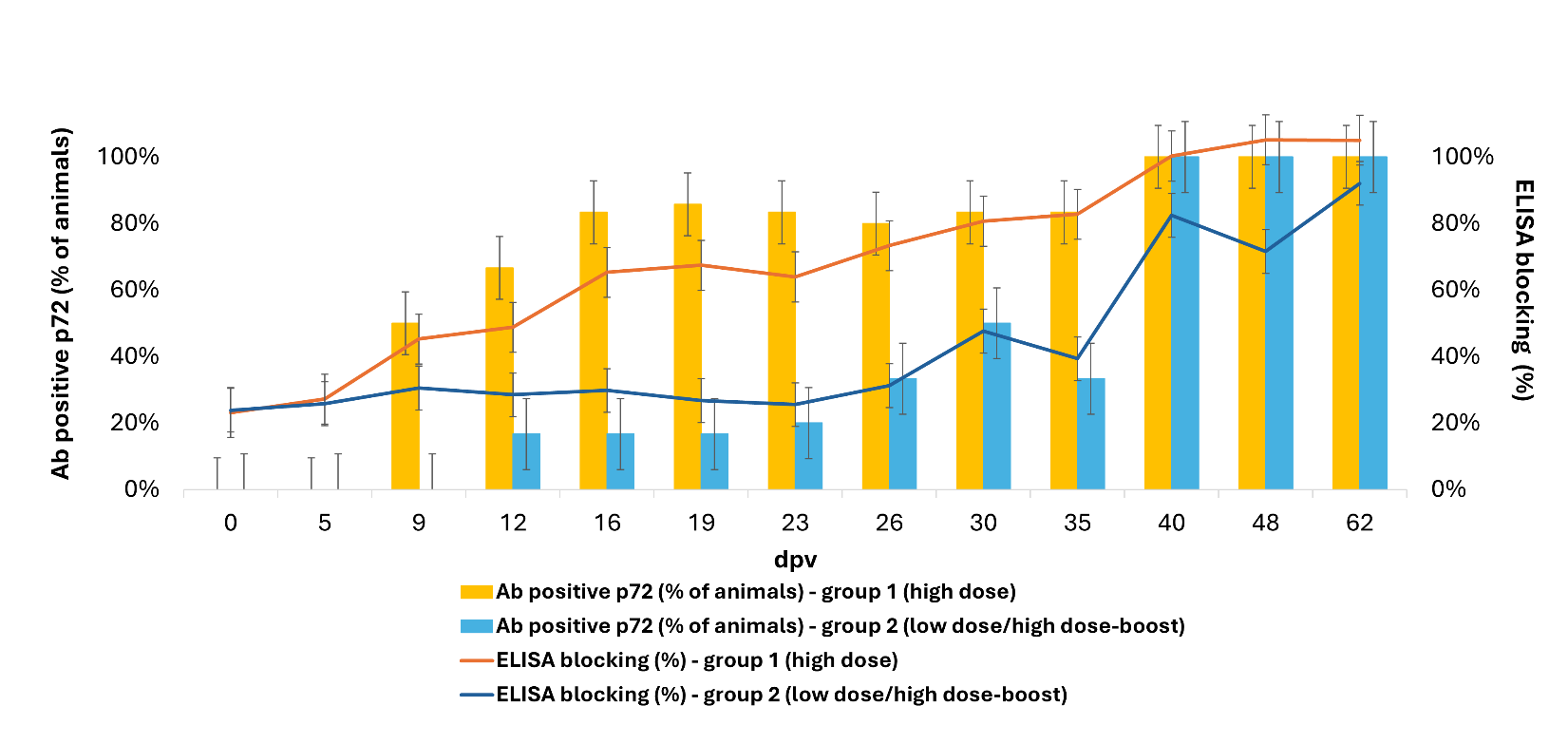
**

**Supplementary Figure 1. Evolution of anti-p72 antibodies and seroconversion rates.** Seroconversion (bars, left Y-axis) and competitive ELISA blocking values (lines, right Y-axis) were monitored over time (dpv). Group 1 received a high-dose double administration; Group 2 received a low-dose/high-dose boost. Error bars represent the standard deviation.

**
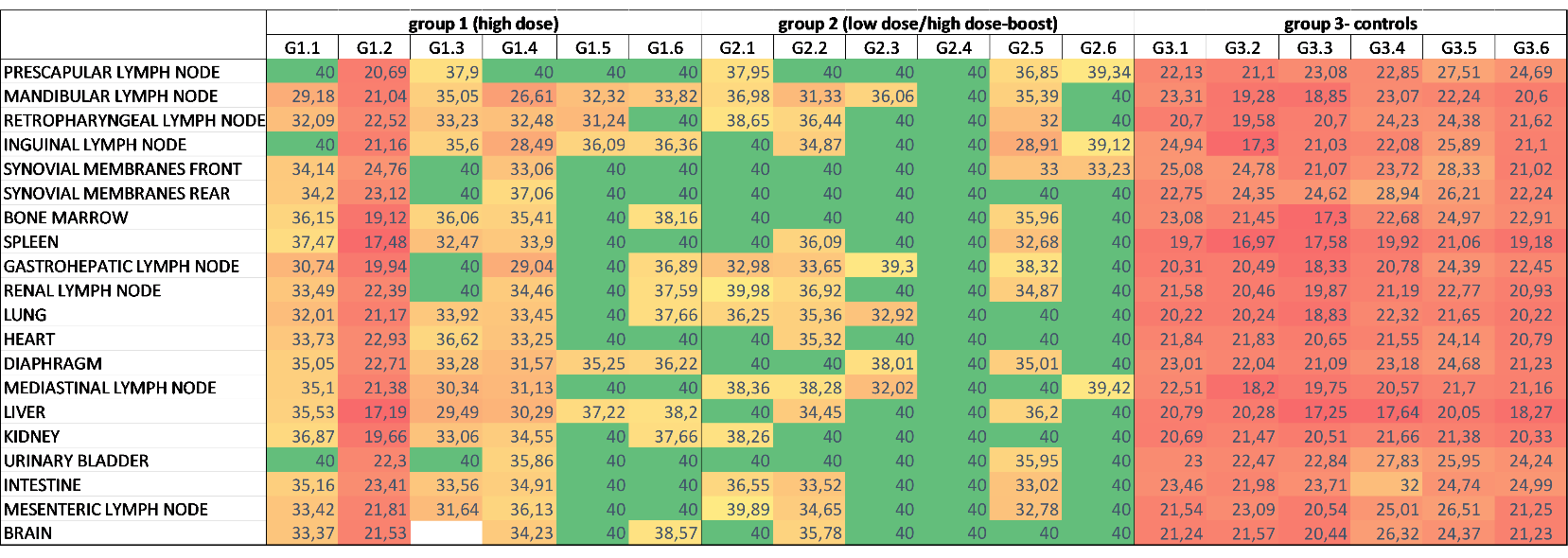
**

**Supplementary Figure 2. Tissue distribution and viral load of ASFV DNA.** Heatmap representing the individual Cq values obtained by qRT-PCR targeting the *p72* gene across various tissues. Individual animals are displayed on the x-axis, categorized into Group 1 (high-dose), Group 2 (low-dose/high-dose-boost), and Group 3 (unvaccinated controls). The color scale illustrates the viral replication gradient: red indicates high viral load (low Cq values), yellow represents intermediate levels, and green signifies low or undetectable viral replication (high Cq values, up to 40).
